# Editorial

**DOI:** 10.1120/jacmp.v5i4.2085

**Published:** 2004-11-24

On behalf of all readers and volunteers, I want to thank and congratulate Dr. Edwin McCullough for his outstanding service to the JACMP and his well‐earned retirement. Ed served as Editor‐in‐Chief for the year 2003 and captained this journal through some stormy seas. In so many ways, we would not have made it without you Ed, so many thanks.

I also want to welcome four new Associate Editors to our team: Nikos Papanikolaou, Lu Wang, Janelle Molloy, and Mehrdad Sarfaraz have joined our editorial team in the area of Radiation Oncology Physics. When you see them, please offer your support and thanks. The JACMP could not exist without the dedicated volunteer effort of our Associate Editors.

Now, let us think back a few years. It is 1998, and members of the American College of Medical Physics are beginning to talk about how to bring a clinical and professional journal into existence. Web‐based technology offered lower costs and a wonderful opportunity to simplify the publication process, but bringing the JACMP into existence raised a number of questions: If the journal is on the Internet, will we need to have a print version? Should we charge a subscription? How do we handle advertising? What is the cost‐and‐expense model? What if papers from the JACMP are posted elsewhere? Who will own the copyright for the articles? Where will we get the software to manage the articles?

It is hard to believe that was seven years ago, and we are completing our fifth year of operation. The JACMP has published 173 clinical and professional papers in addition to book reviews, editorials, etc. There is no subscription; the entire archive is available free to anyone with Web access. There are no page charges or other costs to authors for publication. Authors retain the copyright for their papers and may post these papers anywhere as long as the JACMP is acknowledged. One particularly difficult problem to overcome was the manuscript management software. Products in the marketplace often were not fully featured and were costly to purchase and to maintain. By 2003, it was clear that the JACMP would not exist unless another solution could be found. But what a solution we did find, and that is the story of the Public Knowledge Project (PKP). The PKP is dedicated to exploring whether and how new technologies can be used to improve the professional and public value of scholarly research. It is dedicated to maintaining the public accessibility and coherence of research knowledge in a sustainable and globally accessible form.

The PKP is an initiative of the University of British Columbia, funded by the Canadian government. The PKP has developed the Open Journal Systems (OJS), a journal management and publishing system designed to expand and improve access to research. The OJS is open‐source software made freely available to journals worldwide for making open‐access publishing a viable option for more journals. The developers of PKP believe open access can increase a journal's readership as well as contribute to the public good on a global scale.

It was hard to believe at first. In August 2003, just at the time the JACMP had to come up with a low‐cost solution, a version of OJS was released that contained all the features required to support the Journal. In addition, the OJS has an ongoing support team at the University of British Columbia. Our interaction with John Willinsky and his fine group of supporters resulted in a few bug fixes, some new features, and the ability to begin double‐blind manuscript reviews. Oh, and the price is very right—no cost; that allows us to keep the JACMP free for you to use and enjoy.

Michael D. Mills

Editor‐in‐Chief

## The Journal of Applied Clinical Medical Physics

**Table:** 

**Editorial Board 2004**	
Editor‐in‐Chief	Michael D. Mills
Deputy Editor‐in‐Chief	Timothy D. Solberg
Associate Editor‐at‐Large	Richard Stark
**Associate Editors**	
Radiation Oncology Physics	Nzhde Agazaryan, B. Gino Fallone
	John Gibbons, Michael Herman
	Lu Wang, Edwin McCullough
	Janelle Molloy, Timothy Paul
	Matthew Podgorsak, Nikos Papanikolaou
	Mehrdad Sarfaraz
Medical Imaging	Walter Huda, William Pavlicek
Radiation Measurements	Larry DeWerd
Radiation Protection & Regulations	James Deye
Nonionizing Topics	Bhudatt Paliwal
Other Topics	Timothy Solberg
Book/Media Reviews	James Smathers
Editorials	John Horton
**Journal Production Team**	
Manuscript Manager	Patricia Walker
Copy Editor	Betty R. Robinson
Layout Editor	Frances P. Robinson
Proofreader	Heather Shand

